# Late combination of transarterial chemoembolization with apatinib and camrelizumab for unresectable hepatocellular carcinoma is superior to early combination

**DOI:** 10.1186/s12885-022-09451-1

**Published:** 2022-03-27

**Authors:** Shuguang Ju, Chen Zhou, Junwen Hu, Yingliang Wang, Chaoyang Wang, Jiacheng Liu, Chongtu Yang, Songjiang Huang, Tongqiang Li, Yang Chen, Yaowei Bai, Wei Yao, Bin Xiong

**Affiliations:** 1 Department of Radiology, Union Hospital, Tongji Medical College, Huazhong University of Science and Technology, Wuhan, 430022 China; 2 Hubei Province Key Laboratory of Molecular Imaging, Wuhan, 430022 China; 3 Department of Oncology, The Third People’s Hospital of Yibin, Sichuan, 644000 China

**Keywords:** Apatinib, Immunotherapy, Transarterial chemoembolization, Hepatocellular carcinoma, Prognosis

## Abstract

**Objective:**

The purpose of this study was to explore the efficacy and safety of transarterial chemoembolization (TACE) combined with apatinib and camrelizumab (TACE + AC) for unresectable hepatocellular carcinoma (HCC), and the impact of the timing of the combination on it.

**Methods:**

In this single-arm retrospective study, consecutive data of patients with unresectable HCC treated to our hospital from March 2017 to September 2021 were collected. These patients were treated with TACE and started on camrelizumab and apatinib within one week of TACE. Camrelizumab 200 mg intravenously once every three weeks and apatinib 250 mg orally once daily. Repeat TACE treatment was available on an on-demand basis. The primary endpoints were overall survival (OS) and progression-free survival (PFS). Secondary endpoints included objective response rate (ORR), disease control rate (DCR), and safety. The univariate and multivariate Cox regression analyses were used to assess the effect of early and late combination on OS and PFS.

**Results:**

A total of 80 patients were enrolled in this study. The median OS was 22.1 months (95% confidence interval [CI]: 13.8–30.5 months) and the median PFS was 15.7 months (95% CI: 14.7–16.6 months). The ORR was 58.8% (95% CI: 47.2–69.6) and DCR reached 81.2% (95% CI: 71.0–89.1). Multivariable Cox proportional hazard regression analyses showed that TACE late combined with apatinib and camrelizumab provided better OS than early combination (HR = 0.175, 95% CI:0.060–0.509, *P* = 0.001), as did PFS (HR = 0.422, 95% CI:0.184–0.967, *P* = 0.041). All treatment-related adverse events were tolerable, and no serious adverse events were observed.

**Conclusion:**

TACE combined with apatinib plus camrelizumab for patients with unresectable HCC has promising antitumor activity and a manageable safety profile. For unresectable HCC with large tumor burden, late combination provides better OS and PFS compared to early combination.

## Introduction

Liver cancer is a common cancer and the fourth leading cause of cancer-related death worldwide [1]. Hepatocellular carcinoma (HCC) is the most common type of liver cancer [2]. Hepatitis B virus (HBV) infection is the most predominant risk factor for the development of HCC, and China has a high incidence of HBV infection [3]. Early-stage HCC can be eradicated by surgery, liver transplantation, and ablative therapy, yet most patients with HCC were detected at an advanced stage, losing the chance of radical treatment, and have a poor prognosis [4, 5].

Transarterial Chemoembolization (TACE) is the most commonly performed treatment for unresectable HCC and is recommended as the standard of care for Barcelona Clinic Liver Cancer (BCLC) stage B HCC [4, 6, 7]. In China, different from other HCC clinical practice guidelines, TACE is still recommended for the treatment of HCC with portal invasion and improves the survival of these patients [5]. Repeated TACE therapy can impair liver function and embolization-induced hypoxia can lead to increased vascular endothelial growth factor (VEGF) and promote tumor recurrence and metastasis [8]. Cai et al. [9] found that TACE combined with sorafenib improved OS at 0.5-year and 1-year OS in HCC patients compared to TACE alone, but did not indicate longer survival times. In addition, the heterogeneity of intermediate and advanced HCC is strong and the long-term outcome is still unsatisfactory, and there is an urgent clinical need for better treatment strategies for it [10].

Anti-angiogenic therapies and immunotherapies have demonstrated clinical benefit in advanced HCC, particularly the combination of the two [11]. The IMBrave150 study showed that atezolizumab in combination with bevacizumab significantly improved patient survival compared to sorafenib, the previous first-line treatment for advanced HCC [11]. Based on the IMBrave150 study, atezolizumab in combination with bevacizumab has been recommended by the National Comprehensive Cancer Network (NCCN) as the first-line perfered treatment option for advanced HCC [12]. Similarly, compared with sorafenib, the ORIENT-32 trial also demonstrated the advantages of combination therapy, possibly due to the synergy between anti-angiogenic drugs and anti-PD-1 antibodies [13]. In addition, the RESCUE trial showed that apatinib in combination with camrelizumab (AC) improved survival in patients with unresectable HCC [14]. In those trials, the majority of participants had previously received local treatments, including TACE.

Several studies have demonstrated that TACE combined with anti-angiogenic therapy and immunotherapy could improve the overall survival (OS) of unresectable HCC [15–17]. The importance of TACE in combination therapy was also supported in our previous study [18]. Finding the timing of the appropriate combination therapy is also beneficial in improving the quality of patient survival [19]. Meng et al. [20] found that in TACE combined with sorafenib for HCC that sorafenib should be given orally early after the first TACE. However, the exploration of the timing of TACE combined with anti-angiogenic drugs and immunotherapy has not been reported. What is the best timing for the apatinib plus camrelizumab – early-stage or late-stage after TACE. This study collected clinical data from unresectable HCC patients who received TACE in combination with apatinib and camrelizumab (TACE + AC) in our hospital to study safety and efficacy. Specifically, this study also compared the timing of the combination of TACE and AC and explored the efficacy of early versus late combination of TACE.

## Materials and methods

### Patients

From March 2017 to September 2021, the clinical data of consecutive patients with unresectable HCC who were treated with TACE in combination with apatinib (Jiangsu Hengrui Medicine Co., Ltd., Jiangsu, China) and camrelizumab (Jiangsu Hengrui Medicine Co., Ltd., Jiangsu, China) therapy, were retrospectively reviewed at our institution.

The main inclusion criteria were as follows: (1) histologically, cytologically or clinically confirmed diagnosis of HCC according to the American Association for the Study of Liver Diseases criteria [6], (2) at least 1 measurable lesion according to modified Response Evaluation Criteria in Solid Tumors (mRECIST, version 1.1), (3) BCLC stage C or stage B unsuitable for radical resection, (4) Eastern Cooperative Oncology Group (ECOG) performance status of 0 or 1, (5) Child–Pugh stage A or B, (6) received at least one cycle of TACE in combination with apatinib plus camrelizumab. The major exclusion criteria included: (1) a history of portal hypertensive gastrointestinal bleeding in the 3 months prior to enrollment, (2) other clinical trial participants, (3) uncomplete data.

The study was conducted in accordance with the Declaration of Helsinki and Good Clinical Practice. The study protocol was approved and agreed by the Ethics Review Committee of Wuhan Union Hospital.

### Treatment protocol

The TACE procedure was performed by interventionalists with over 10 years of experience, including conventional TACE (cTACE) or drug-eluting bead TACE (DEB-TACE), as described in our previous study [18]. The 5 French (F) RH catheter (Cook, Inc., Bloomington, Indiana, USA) was placed into the opening of the common hepatic artery through the femoral artery by the Seldinger method. After DSA angiography showed the lesion region and the tumor supply artery, a 2.7 F microcatheter (Terumo, Tokyo, Japan) was inserted super-selectively into the tumor supply artery. The type of embolism was decided by both the doctor and the patient. For cTACE, embolization was performed using iodine oil mixed with doxorubicin emulsion, and the vascular trunk was embolized with 350–560 μm absorbable gelatin sponge particles (Hangzhou Alicon Pharm SCI & TEC CO., Ltd., Zhejiang, China). For DEB-TACE, embolization was conducted using CalliSpheres (Jiangsu Hengrui Medicine Co., Ltd., Jiangsu, China) of different diameters loaded with 60 mg of doxorubicin. The endpoint of embolization is the stagnation of blood flow in the tumor-supplying artery. TACE was repeated for patients whose organ function and physical status have not deteriorated, if follow-up enhanced CT or MRI showed that the tumor still had a blood supply. Francesco et al. [21] showed that it is important that experienced radiologists evaluate imaging. Therefore, two experienced radiologists evaluated the patient’s CT or MRI images to assess whether the tumor still had an arterial blood supply in this study.

Apatinib and camrelizumab were initiated within 7 days after TACE. Apatinib at a dose of 250 mg was orally administered once a day. Camrelizumab was injected intravenously at 200 mg once every 3 weeks. Apatinib was suspended 3 days before the next TACE treatment. Doses of apatinib and camrelizumab were reduced, suspended, or discontinued when patients experience serious adverse events (AEs).

Early combination was defined as treatment with apatinib and camrelizumab after the first or second TACE. Late combination was defined as having had at least three times TACE before receiving treatment with apatinib and camrelizumab. Timing of apatinib and camrelizumab application was a decision for both physicians and patients.

### Study endpoints

The primary endpoints of this study were OS and progression-free survival (PFS). In addition, we explored whether the timing of TACE in combination with AC was an independent risk factor for prognosis. OS was defined as the time between the start of treatment and death from any cause or last contact. PFS was defined as the time from treatment initiation to the first reported disease progression or death from any cause. The secondary endpoints were objective response rate (ORR) and disease control rate (DCR). Tumor response was evaluated by two experienced radiologists using mRECIST. AEs were graded according to the National Cancer Institute (NCI) Common Terminology Criteria for Adverse Events (CTCAE) version 5.0. Adverse events directly attributable to TACE (e.g., post-embolization syndrome) based on care experience were no longer listed.

### Statistical methods

All statistical analyses were performed by SPSS 23.0 statistical software (IBM Corp., Armonk, NY, USA). Normally distributed continuous variables were expressed using the mean ± standard deviation (SD), and non-normally distributed continuous variables were presented using median (interquartile range). Number (percentage) were used for categorical variables. 95% confidence intervals (95% CI) of ORR and DCR were estimate by Clopper-Pearson method. The median OS and PFS were estimate using the Kaplan–Meier method. Univariable and multivariable Cox proportional hazards were used to measure the factors affecting OS and PFS, and factors with *P* value < 0.1 in univariate analyses were included in the multivariate analyses. A two-sided *P* value < 0.05 was considered statistically significant.

## Results

### Patient characteristics

**Fig. 1:**
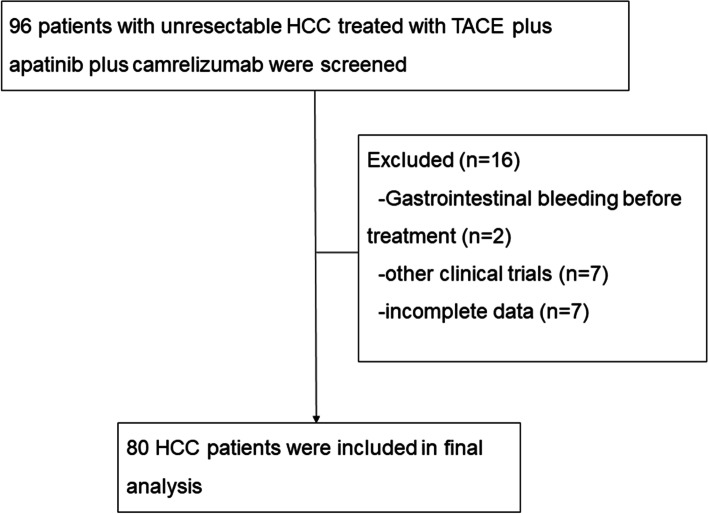
Flow diagram. HCC = hepatocellular carcinoma; TACE = transarterial chemoembolization

A total of 96 patients with unresectable HCC were reviewed, and 80 patients were enrolled in this study between March 2017 and September 2021 (Fig. 1). The median follow-up time is 14.6 months (95% CI: 12.1–17.2 months) as of January 2022. The reasons for exclusion in 16 patients were as follows: gastrointestinal bleeding before treatment (*n* = 2), other clinical trials (*n* = 7), and incomplete data (*n* = 7). The median age was 52 years (range: 46–62), and 66 (82.5%) of the patients were male; forty-six (57%) patients had an ECOG performance status of 1, and 58 (72.5%) patients had a Child–Pugh class of A; forty-five (56.3%) patients were treated early in combination; forty-four (55%) had extrahepatic metastases, 47 (58.8%) had macrovascular invasion, and 67 (83.7%) had a BCLC stage of C; six-five (81.3%) patients had hepatitis B infection and 70 (87.5%) had liver cirrhosis; the tumor size was 9.7 ± 4.7 cm, and 67 (83.8%) patients had multiple tumor distribution (Table 1).

**Table 1:** Baseline characteristics of the patients

Characteristics	TACE plus apatinib plus camrelizumab (*n* = 80)	early combination group (*n* = 45)	late combination group (*n* = 35)
Age (years), median (IQR)	52 (46–62)	53 (48–60)	50 (43–63)
Age group (years)			
< 65	66 (82.5)	37 (82.2)	29 (82.9)
≥ 65	14 (17.5)	8 (17.8)	6 (17.1)
Sex			
Male	66 (82.5)	36 (80.0)	30 (85.7)
Female	14 (17.5)	9 (20.0)	5 (14.3)
ECOG PS			
0	34 (42.5)	15 (33.3)	19 (54.3)
1	46 (57.5)	30 (66.7)	16 (45.7)
Child–Pugh class			
A	58 (72.5)	29 (64.4)	29 (82.9)
B	22 (27.5)	16 (35.6)	6 (17.1)
Co-treatment time			
Early combination	45 (56.3)	45 (100.0)	0 (0.0)
Late combination	35 (43.8)	0 (0.0)	35 (100.0)
Extrahepatic metastases	44 (55.0)	27 (60.0)	17 (48.6)
Macrovascular invasion	47 (58.8)	30 (66.7)	17 (48.6)
BCLC stage			
B	13 (16.3)	4 (8.9)	9 (25.7)
C	67 (83.7)	41 (91.1)	26 (74.3)
Liver Cirrhosis	70 (87.5)	38 (84.4)	32 (91.4)
Hepatitis B	65 (81.3)	35 (77.8)	30 (85.7)
Tumor distribution			
Single	13 (16.3)	7 (15.6)	6 (17.1)
Multiple	67 (83.8)	38 (84.4)	29 (82.9)
Tumor size (cm, mean ± SD)	9.7 ± 4.7	10.0 ± 4.5	9.3 ± 5.0
< 10 cm	45 (56.3)	25 (55.6)	20 (57.1)
≥ 10 cm	35 (43.8)	20 (44.4)	15 (42.9)
Laboratory parameters			
RBC (10^9/L, mean ± SD)	4.1 ± 0.8	4.0 ± 0.8	4.2 ± 0.9
Hb (g/L), median (IQR)	128 (110–138)	129 (112–136)	128 (110–139)
Platelet (10^9/L), median (IQR)	163 (119–228)	151 (111–210)	181 (144–372)
WBC (10^12/L, mean ± SD)	5.8 ± 1.9	5.5 ± 1.8	6.3 ± 2.0
Neutrophils (10^9/L, mean ± SD)	3.9 ± 1.7	3.6 ± 1.4	4.2 ± 1.9
Lymphocyte (10^9/L, mean ± SD)	1.3 ± 0.5	1.2 ± 0.5	1.4 ± 0.5
NLR, median (IQR)	2.8 (2.0–4.5)	2.9 (2.2–4.6)	2.8 (1.8–4.4)
ALT (U/L), median (IQR)	38 (25–57.5)	38.5 (25–58)	35 (25–56.5)
AST (U/L), median (IQR)	46 (34.5–84.5)	61.5 (39–105)	41 (33.5–54)
TBIL (μmmol/L), median (IQR)	14.4 (10.8–19)	15.2 (10.8–21.7)	13.4 (10.7–17.4)
ALP (U/L), median (IQR)	134 (102–203.5)	147 (109–224)	118 (89.5–169.5)
TBA (μmol/L), median (IQR)	7.8 (4.1–14.3)	8.35 (4.1–17)	7.7 (4.5–13.1)
ALB (g/L, mean ± SD)	36.4 ± 4.9	35.2 ± 4.1	38.0 ± 5.3
AFP (ng/mL)			
< 200	28 (35.0)	14 (31.1)	14 (12.3)
≥ 200	52 (65.0)	31 (68.9)	21 (60.0)

Data are median (range) or N (%). *TACE* transcatheter arterial chemoembolization

*ECOG PS* Eastern Cooperative Oncology Group Performance Status, *BCLC* Barcelona Clinic Liver Cancer, *RBC* red blood cell, *Hb* Hemoglobin, *WBC* white blood cell, *NLR* Neutrophils/ Lymphocyte, *ALT* Alanine aminotransferase, *AST* Aspartate aminotransferase, *TBIL* total bilirubin, *ALP* alkaline phosphatase, *TBA* total bile acid, *ALB* albumin, *AFP* alpha-fetoprotein

Early combination was defined as treatment with apatinib and camrelizumab after the first or second TACE. Late combination was defined as having had at least three times TACE before receiving treatment with apatinib and camrelizumab

### Efficacy

**Fig. 2:**
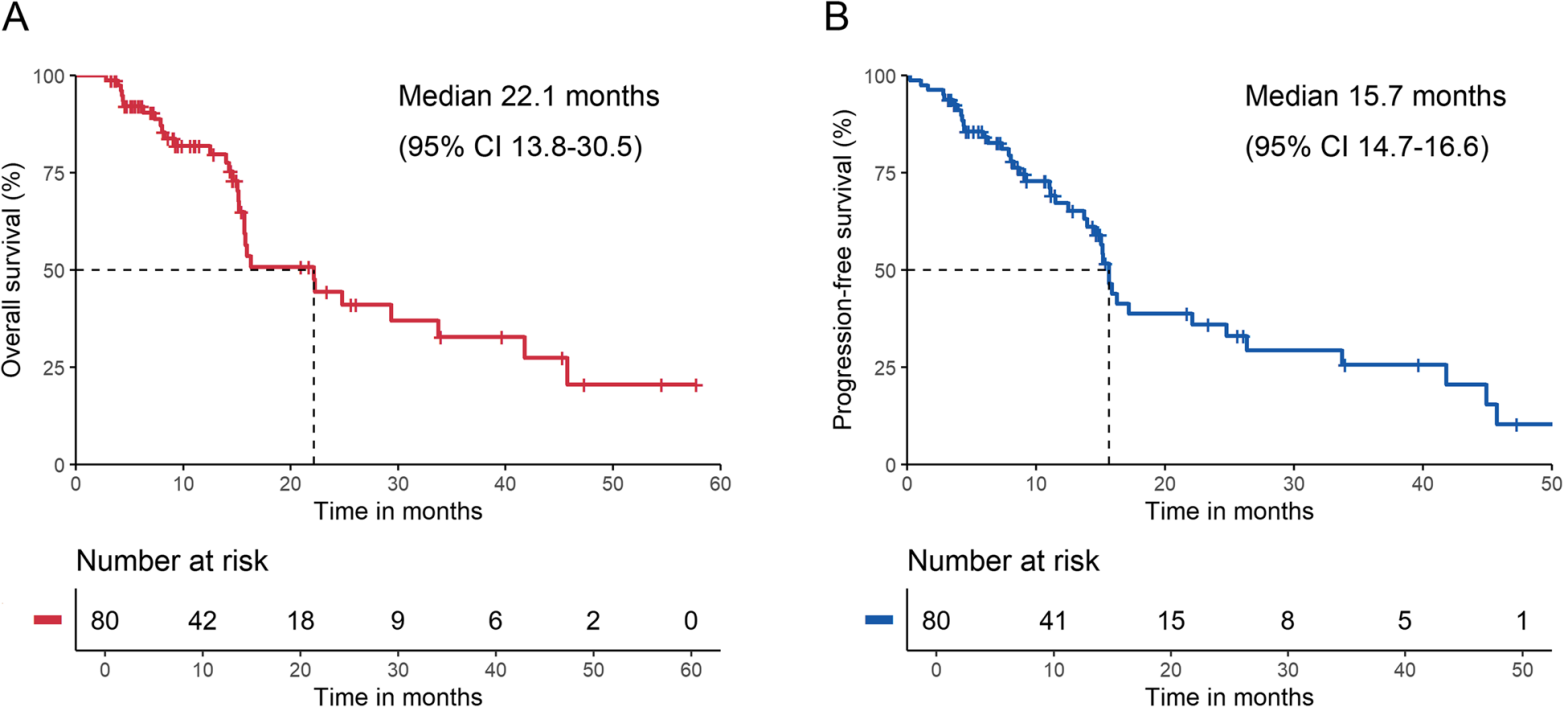
Kaplan–Meier plots of overall survival (**A**) and progression-free survival (**B**)

**Fig. 3:**
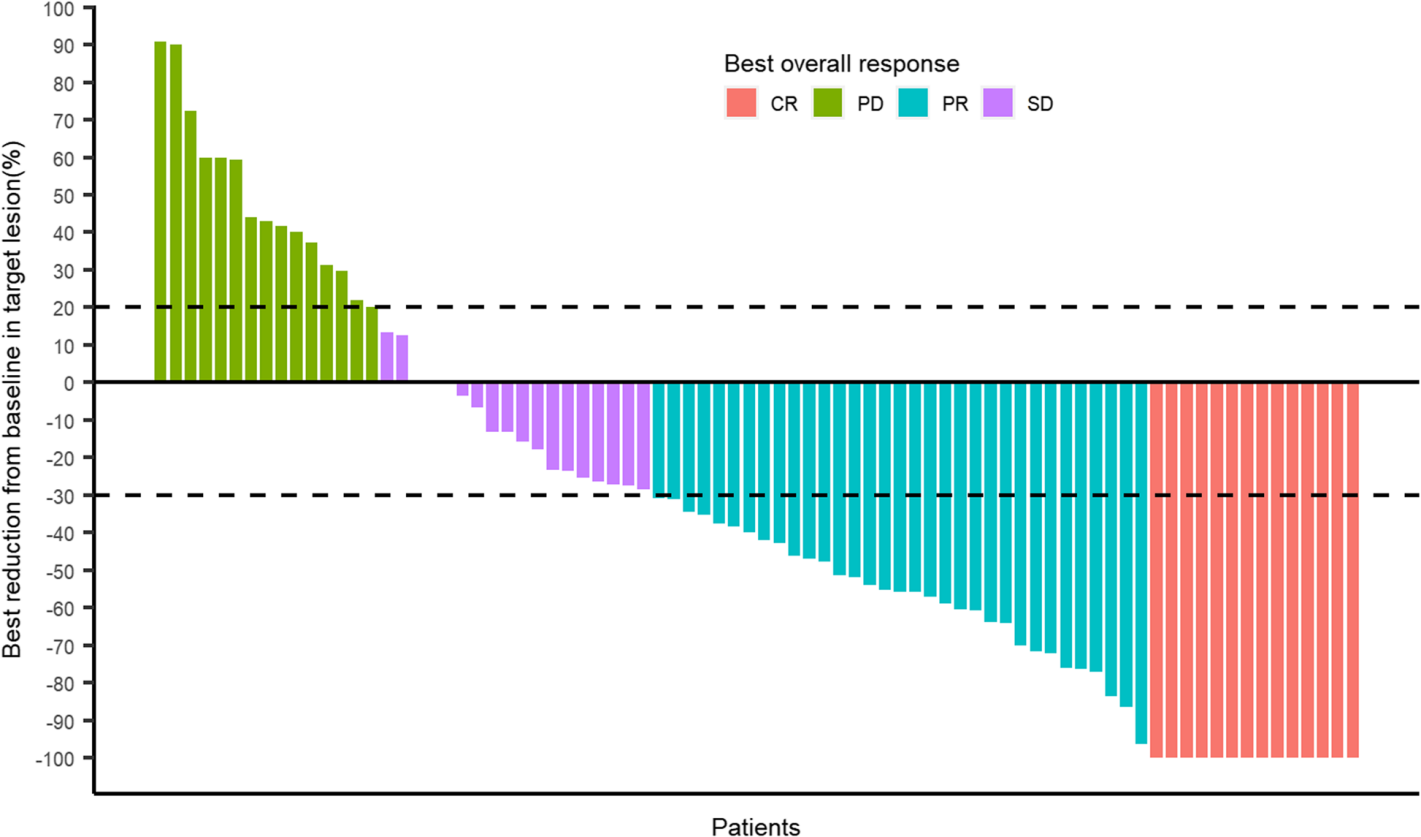
The best change from baseline in sum of the target lesion diameter per patient. CR = complete response; PR = partial response; SD = stable disease; PD = progressive disease

All 80 patients were included in the efficacy analysis. In this study, the median OS of patients with unresectable HCC treated with TACE + AC was 22.1 months (95% CI: 13.8–30.5 months) (Fig. 2A), and the median PFS was 15.7 months (95% CI: 14.7–16.6 months) (Fig. 2B). On the basis of mRECIST 1.1, 47 patients had an objective response (58.8% [95% CI: 47.2–69.6]), and 65 patients had disease control (81.2% [95% CI: 71.0–89.1]) (Table 2). Sixty (75%) patients had a reduction in target lesion load compared to baseline (Fig. 3).

**Table 2:** Tumor response

	TACE plus apatinib plus camrelizumab (*n* = 80)
Best overall response	
Complete response (CR)	14 (17.5)
Partial response (PR)	33 (41.3)
Stable disease (SD)	18 (22.5)
Progressive disease (PD)	15 (18.7)
ORR (CR + PR)	47 (58.8, 47.2–69.6)
DCR (CR + PR + SD)	65 (81.2, 71.0–89.1)

Data are N (%)

*DCR* disease control rate, *ORR* objective response rate, *TACE* transarterial chemoembolization

Based on the results of univariable and multivariable Cox proportional hazard regression analysis, age group (≥ 65 vs. < 65; hazard ratio [HR] = 2.545, 95% CI: 1.113–5.823, *P* = 0.027), tumor size (HR = 1.010, 95% CI: 1.003–1.017, *P* = 0.005), and co-treatment time (late combination vs. early combination; HR = 0.175, 95% CI:0.060–0.509, *P* = 0.001) were confirmed as the independent prognostic factors for OS. Meanwhile, macrovascular invasion (Yes vs. No; HR = 2.193, 95% CI:1.083–4.443, *P* = 0.029), co-treatment time (late combination vs. early combination; HR = 0.422, 95% CI:0.184–0.967, *P* = 0.041), and ALP were identified as the independent prognostic factors for PFS (Table 3).

**Table 3:** Analyses of prognostic factors for survival

	OS				PFS			
	Univariate analysis		Multivariate analysis		Univariate analysis		Multivariate analysis	
	HR (95%CI)	*P*	HR (95%CI)	*P*	HR (95%CI)	*P*	HR (95%CI)	*P*
Sex (female VS male)	0.639 (0.222–1.835)	0.405			1.284 (0.534–3.086)	0.576		
Age (per 1 point increase)	1.027 (0.993–1.063)	0.125			1.001 (0.972–1.030)	0.953		
Age group (≥ 65 VS < 65)	2.467 (1.153–5.279)	0.020	2.545 (1.113–5.823)	0.027	1.507 (0.734–3.094)	0.264		
ECOG PS (per 1 point increase)	1.363 (0.664–2.802)	0.399			1.679 (0.891–3.164)	0.109		
BCLC stage (C VS B)	2.185 (0.829–5.756)	0.114			1.922 (0.843–4.379)	0.120		
Tumor size (per 1 point increase)	1.010 (1.003–1.017)	0.003	1.010 (1.003–1.017)	0.005	1.006 (1.000–1.012)	0.052	-	-
Tumor size (≥ 10 cm VS < 10 cm)	2.200 (1.050–4.613)	0.037	-	-	1.523 (0.803–2.890)	0.198		
Tumor distribution (multiple VS single)	2.190 (0.663–7.233)	0.199			1.890 (0.668–5.344)	0.230		
Child–Pugh class (B VS A)	1.927 (0.878–4.232)	0.102			1.913 (0.965–3.791)	0.063	-	-
Extrahepatic metastases (Yes VS No)	1.638 (0.796–3.367)	0.180			1.412 (0.757–2.636)	0.278		
Macrovascular invasion (Yes VS No)	2.533 (1.151–5.574)	0.021	-	-	2.332 (1.174–4.632)	0.016	2.193 (1.083–4.443)	0.029
Liver Cirrhosis (Yes VS No)	0.444 (0.126–1.563)	0.206			0.634 (0.218–1.843)	0.402		
Hepatitis B (Yes VS No)	0.562 (0.210–1.504)	0.251			0.965 (0.372–2.501)	0.942		
Co-treatment time (late combination VS early combination)	0.200 (0.077–0.522)	0.001	0.175 (0.060–0.509)	0.001	0.310 (0.144–0.665)	0.003	0.422 (0.184–0.967)	0.041
AFP (≥ 200 VS < 200)	1.946 (0.892–4.244)	0.094	-	-	1.846 (0.937–3.636)	0.076	-	-
RBC	0.956 (0.599–1.526)	0.956			0.880 (0.590–1.312)	0.529		
WBC	0.941 (0.769–1.153)	0.559			0.892 (0.740–1.075)	0.229		
Platelet	1.004 (1.000–1.007)	0.065	-	-	1.002 (0.999–1.006)	0.169		
Hb	0.993 (0.977–1.011)	0.451			0.990 (0.976–1.005)	0.189		
Neutrophils	1.018 (0.829–1.249)	0.866			0.967 (0.803–1.166)	0.728		
Lymphocyte	0.377 (0.166–0.854)	0.019	-	-	0.410 (0.200–0.842)	0.015	-	-
NLR	1.074 (0.916–1.259)	0.379			1.078 (0.938–1.240)	0.288		
ALT	1.004 (0.995–1.014)	0.362			1.003 (0.996–1.010)	0.372		
AST	1.004 (0.998–1.009)	0.189			1.003 (0.999–1.008)	0.130		
TBIL	1.006 (0.973–1.040)	0.716			1.002 (0.971–1.034)	0.901		
ALP	1.005 (1.002–1.008)	0.002	-	-	1.004 (1.002–1.007)	0.002	1.004 (1.001–1.007)	0.017
TBA	0.994 (0.982–1.007)	0.348			1.000 (0.994–1.006)	0.948		
ALB	0.949 (0.884–1.018)	0.142			0.948 (0.892–1.008)	0.088	-	-

*ECOG PS* Eastern Cooperative Oncology Group Performance Status, *BCLC* Barcelona Clinic Liver Cancer, *RBC* red blood cell, *Hb* Hemoglobin, *WBC* white blood cell, *NLR* Neutrophils/ Lymphocyte, *ALT* Alanine aminotransferase, *AST* Aspartate aminotransferase, *TBIL* total bilirubin, *ALP* alkaline phosphatase, *TBA* total bile acid, *ALB* albumin, *AFP* alpha-fetoprotein

Early combination was defined as treatment with apatinib and camrelizumab after the first or second TACE. Late combination was defined as having had at least three times TACE before receiving treatment with apatinib and camrelizumab

### Safety

Safety analyses were conducted in 80 enrolled patients. The most common AEs of any grade associated with apatinib and camrelizumab include hand-foot skin reaction, hypertension, proteinuria, fatigue, reactive cutaneous capillary endothelial proliferation (RCCEP), and abnormal thyroid function. Among the patients, there were 33 cases (41.3%) of hand-foot skin reaction, 35 cases (43.8%) of hypertension, 25 cases (31.1%) of proteinuria, 16 cases (20.0%) of fatigue, 16 cases (20.0%) of RCCEP, 18 cases (22.6%) of abnormal thyroid function. The grade 3–4 treatment-related AEs that occurred were hand-foot skin reaction in 5 patients (6.3%), hypertension in 6 patients (7.5%), proteinuria in 1 patient (1.3%), fatigue in 2 patients (2.5%), diarrhoea in 2 patients (2.5%), and myocarditis in 1 patient (1.3%) (Table 4). As a result, these 16 patients required a dose reduction of apatinib and another patient discontinued subsequent use of camrelizumab due to myocarditis. Adverse events in all patients were effectively controlled by symptomatic treatment or reduction in drug dose or discontinuation, and there were no treatment-related deaths.

**Table 4:** Treatment-related adverse events

n (%)	TACE plus apatinib plus camrelizumab (*n* = 80)	
	All grade	Grade ≥ 3
Hand-foot skin reaction	33 (41.3)	5 (6.3)
Hypertension	35 (43.8)	6 (7.5)
Fatigue	16 (20.0)	2 (2.5)
Mouth ulcers	5 (6.3)	0 (0)
Proteinuria	25 (31.3)	1 (1.3)
Rash	11 (13.8)	0 (0)
Hoarseness	4 (5.0)	0 (0)
Gingival hamorrhage	3 (3.8)	0 (0)
Decreased appetite	14 (17.5)	0 (0)
Diarrhoea	13 (16.3)	2 (2.5)
Hypothyroidism	15 (18.8)	0 (0)
Hyperthyroidism	3 (3.8)	0 (0)
RCCEP	16 (20.0)	0 (0)
Myocarditis	3 (3.8)	1 (1.3)
Haemorrhage, upper GI	3 (3.8)	0 (0)

*RCCEP* reactive cutaneous capillary endothelial proliferation, *GI* gastrointestinal tract

## Discussion

Despite significant developments in screening tools that have led to an increase in the detection of early HCC [22], the majority of patients are still diagnosed with unresectable HCC at the time of initial diagnosis [5]. In recent years, treatment options for unresectable HCC have evolved rapidly. The IMBrave 150 trial demonstrated better overall and progression-free survival with atezolizumab in combination with bevacizumab than sorafenib [11]. Atezolizumab combined with bevacizumab was recommended as the first-line treatment of choice for advanced HCC, validating the clinical benefits of anti-angiogenic therapy in combination with anti-PD-1 antibodies for unresectable HCC in clinical practice [12]. The RESCUE trial, an open-label, phase II clinical trial, showed that apatinib in combination with camrelizumab in first- or second-line treatment of advanced HCC exhibits favorable efficacy and safety, providing a new treatment option for unresectable HCC [14]. Most patients had received local treatment including TACE [11, 14], but there are fewer studies on TACE combined with anti-angiogenic therapy and anti-PD-1 antibodies.

This study evaluated the efficacy and safety of TACE + AC for unresectable HCC and explored the factors affecting its prognosis. In this study, patients had a median OS of 22.1 months (95% CI: 13.8–30.5 months) and a median PFS of 15.7 months (95% CI: 14.7–16.6 months), which were similar to previous studies [16, 17]. Cao et al. [16] found median OS and PFS of 23.6 and 13.3 months for TACE combined with lenvatinib and sintilimab for unresectable HCC, respectively. Liu et al. [17] showed that median OS and PFS of 24 and 11.4 months for TACE combined with lenvatinib and camrelizumab for unresectable HCC, respectively. On the other hand, the ORR of 58.8% (95% CI: 47.2–69.6) and DCR of 81.2% (95% CI: 71.0–89.1) in this study were superior to the ORR (34.3% [95%CI: 23.3–46.6]) and DCR (77.1% [95% CI: 65.6–86.3]) of the RESCUE trial, which may be due to the fact that only 60% of patients in the RESCUR trial had received local treatment [14]. The results showed that TACE + AC therapy significantly improved the survival of patients with unresectable HCC. One possible reason is that TACE causes ischemic necrosis of the tumor and reduces tumor load, which increases the release of tumor antigens and the increase in PD-1 and PD-L1 expression, improving tumor recognition [23]. Another possible reason is that TACE has altered the tumor microenvironment to produce a more inflammatory environment, which may support a better T-cell response [24]. Third, anti-VEGF therapy normalizes tumor vasculature and additionally reduces VEGF-mediated immunosuppression in tumors and their microenvironment and may enhance the efficacy of anti-PD-1 and anti-programmed death ligand 1 (PD-L1) by reversing VEGF-mediated immunosuppression and promoting T-cell infiltration in tumors [11, 25, 26]. Therefore, the combination of TACE, apatinib and camrelizumab may lead to synergistic antitumor effects and improved clinical outcomes in patients with unresectable HCC.

The results of multivariable Cox proportional hazard regression analysis showed that the timing of TACE and drug combination therapy was an independent risk factor for OS and PFS. Previous studies have also shown that sequential use of immune checkpoint inhibitors after radiotherapy improves patient survival compared to concurrent use in other tumors [27, 28]. Late combination had longer survival and progression-free survival compared to early combination, which may be due to the following reasons: (1) The tumor microenvironment in advanced HCC is in an immunosuppressed state, especially in patients with co-infection with chronic hepatitis B. T cells are dysfunctional. TACE therapy could improve this state by producing a more inflammatory environment that is more suitable for T-cell responses [24, 29]. (2) The process of tumor ischemic-hypoxic necrosis releasing large amounts of antigens and altering the tumor microenvironment after TACE are time-consuming. (3) Early-stage tumors may be better treated with immunotherapy [30], while advanced HCC can be downstaged and reduce tumor burden after multiple TACE treatments to achieve similar results as early-stage tumors [31]. In addition, there is a major issue of choosing a repeat treatment for TACE and the optimal number of TACEs before switching to another treatment or best supportive care [32]. Although an ART score is available to determine whether a patient needs repeat TACE therapy [33], unfortunately, this ART score is not appropriate for all HCC patients [32]. Therefore, more research is still needed find the optimal timing of TACE in combination with AC and the optimal number of TACE, and to explain and validate this situation in order to guide clinical practice to provide more clinical benefit to the HCC patients.

The majority of AEs observed with the combination of apatinib and camrelizumab on top of TACE were consistent with the safety of the drugs alone [14, 34]. No new or unexpected adverse events were observed. For apatinib, the majority of AEs were hand-foot skin reaction, hypertension, proteinuria, and fatigue. For camrelizumab, the common AEs were RCCEP and abnormal thyroid function. Notably, the incidence of RCCEP was reduced in combination therapy compared to that in camrelizumab monotherapy, which may be due to the involvement of the VEGF signaling pathway in the mechanism of RCCEP, and similar findings have been reported in other studies [14, 35]. More than 80% of the patients in this study had hepatitis B infection combined with cirrhosis, while the incidence of gastrointestinal bleeding was 3.8%, indicating that combination therapy did not increase the risk of gastrointestinal bleeding. In addition, the TACE procedures in this study were performed by experienced interventional radiologists, which reduced the stochastic and non-stochastic risk of x-ray exposure [36]. Generally, the AEs in patients were tolerable, and no deaths due to toxicity occurred, indicating an acceptable safety profile for TACE + AC.

This study had several limitations. First, this study was a single-arm, single-center retrospective study with a relatively small sample size, which may reduce the statistical power. Second, the follow-up period of this study was relatively short, and longer follow-up is needed to verify further survival and progression-free survival. Third, the combination timing grouping applied in this study may be biased, and the appropriate timing of combination therapy still needs to be found. Therefore, prospective, future prospective, multicenter, randomized controlled clinical trials are needed to evaluate the safety and efficacy of TACE + AC in the treatment of unresectable HCC and to find the optimal timing of combination therapy.

## Conclusions

In conclusion, TACE in combination with apatinib and camrelizumab demonstrated encouraging antitumor activity and a manageable safety profile in the treatment of unresectable HCC, providing a feasible and well-tolerated treatment option for these patients. For unresectable HCC with large tumor burden, the survival of TACE late combined with AC is better than early combination, which provides a new idea to explore the optimal timing of TACE combined with anti-angiogenic therapy plus immunotherapy.

## Data Availability

The datasets used and analysed during the current study are available from the corresponding author on reasonable request.
